# Supplementary material for: Modeling glioblastoma heterogeneity as a dynamic network of cell states
Source: Mol Syst Biol. 2021 Sep 16;17(9):e10105. doi: 10.15252/msb.202010105 (PMC8444284; doi:10.15252/msb.202010105)
Supplement: Supplementary file 6 — Source Data for Figure 5 [file MSB-17-e10105-s004.zip › Figure5A_sourcedata/GSEA_3017/nosigdb_stateF.GseaPreranked.1622795015644/NB_KI_CLUSTER5.html]

Details for gene set NB\_KI\_CLUSTER5[GSEA]

|  || Dataset | state33017 |
| Phenotype | NoPhenotypeAvailable |
| Upregulated in class | na\_neg |
| GeneSet | NB\_KI\_CLUSTER5 |
| Enrichment Score (ES) | -0.84251624 |
| Normalized Enrichment Score (NES) | -5.16573 |
| Nominal p-value | 0.0 |
| FDR q-value | 0.0 |
| FWER p-Value | 0.0 |
Table: GSEA Results Summary

  

Fig 1: Enrichment plot: NB\_KI\_CLUSTER5      
 Profile of the Running ES Score & Positions of GeneSet Members on the Rank Ordered List

  

| PROBE | GENE SYMBOL | GENE\_TITLE | RANK IN GENE LIST | RANK METRIC SCORE | RUNNING ES | CORE ENRICHMENT || 1 | GAPDH |  |  | 1361 | 0.261 | -0.6215 | No |
| 2 | MCM3 |  |  | 1738 | -0.335 | -0.7892 | No |
| 3 | RAN |  |  | 1772 | -0.350 | -0.7991 | No |
| 4 | LIG1 |  |  | 1797 | -0.361 | -0.8046 | No |
| 5 | FANCA |  |  | 1844 | -0.385 | -0.8199 | No |
| 6 | SHCBP1 |  |  | 1886 | -0.412 | -0.8325 | No |
| 7 | MXD3 |  |  | 1905 | -0.428 | -0.8342 | No |
| 8 | DIAPH3 |  |  | 1924 | -0.439 | -0.8358 | Yes |
| 9 | GINS2 |  |  | 1932 | -0.445 | -0.8323 | Yes |
| 10 | MZT1 |  |  | 1936 | -0.447 | -0.8269 | Yes |
| 11 | DDX11 |  |  | 1952 | -0.456 | -0.8269 | Yes |
| 12 | CDT1 |  |  | 1961 | -0.460 | -0.8236 | Yes |
| 13 | ZWILCH |  |  | 1962 | -0.461 | -0.8165 | Yes |
| 14 | DNMT1 |  |  | 1969 | -0.465 | -0.8122 | Yes |
| 15 | NASP |  |  | 1988 | -0.481 | -0.8132 | Yes |
| 16 | ARL6IP1 |  |  | 2001 | -0.494 | -0.8112 | Yes |
| 17 | CHEK1 |  |  | 2013 | -0.504 | -0.8086 | Yes |
| 18 | RANBP1 |  |  | 2024 | -0.517 | -0.8054 | Yes |
| 19 | HELLS |  |  | 2038 | -0.528 | -0.8033 | Yes |
| 20 | TRIP13 |  |  | 2044 | -0.530 | -0.7976 | Yes |
| 21 | SPDL1 |  |  | 2064 | -0.569 | -0.7977 | Yes |
| 22 | ZWINT |  |  | 2069 | -0.577 | -0.7907 | Yes |
| 23 | CEP152 |  |  | 2070 | -0.578 | -0.7820 | Yes |
| 24 | RACGAP1 |  |  | 2072 | -0.579 | -0.7736 | Yes |
| 25 | BUB3 |  |  | 2078 | -0.596 | -0.7669 | Yes |
| 26 | GGH |  |  | 2080 | -0.603 | -0.7582 | Yes |
| 27 | CENPK |  |  | 2082 | -0.607 | -0.7494 | Yes |
| 28 | PLK1 |  |  | 2087 | -0.624 | -0.7418 | Yes |
| 29 | NCAPD2 |  |  | 2089 | -0.627 | -0.7327 | Yes |
| 30 | CKS2 |  |  | 2115 | -0.694 | -0.7336 | Yes |
| 31 | ANP32B |  |  | 2119 | -0.700 | -0.7244 | Yes |
| 32 | CENPM |  |  | 2125 | -0.709 | -0.7159 | Yes |
| 33 | CKAP2 |  |  | 2131 | -0.729 | -0.7071 | Yes |
| 34 | CIP2A |  |  | 2132 | -0.733 | -0.6960 | Yes |
| 35 | BRCA1 |  |  | 2138 | -0.758 | -0.6868 | Yes |
| 36 | H2AFV |  |  | 2143 | -0.779 | -0.6768 | Yes |
| 37 | FBXO5 |  |  | 2144 | -0.779 | -0.6650 | Yes |
| 38 | GMNN |  |  | 2146 | -0.785 | -0.6535 | Yes |
| 39 | DEK |  |  | 2154 | -0.817 | -0.6443 | Yes |
| 40 | DUT |  |  | 2155 | -0.824 | -0.6318 | Yes |
| 41 | NUCKS1 |  |  | 2158 | -0.832 | -0.6201 | Yes |
| 42 | MCM7 |  |  | 2160 | -0.839 | -0.6078 | Yes |
| 43 | TUBB4B |  |  | 2162 | -0.843 | -0.5955 | Yes |
| 44 | ECT2 |  |  | 2163 | -0.846 | -0.5826 | Yes |
| 45 | KIF20B |  |  | 2165 | -0.852 | -0.5701 | Yes |
| 46 | LBR |  |  | 2173 | -0.870 | -0.5601 | Yes |
| 47 | KPNA2 |  |  | 2178 | -0.894 | -0.5484 | Yes |
| 48 | CHAF1A |  |  | 2182 | -0.903 | -0.5361 | Yes |
| 49 | MIS18BP1 |  |  | 2183 | -0.903 | -0.5224 | Yes |
| 50 | PRR11 |  |  | 2187 | -0.930 | -0.5096 | Yes |
| 51 | CENPW |  |  | 2192 | -0.943 | -0.4971 | Yes |
| 52 | TUBA1B |  |  | 2193 | -0.947 | -0.4828 | Yes |
| 53 | UBE2T |  |  | 2196 | -0.965 | -0.4690 | Yes |
| 54 | CKS1B |  |  | 2201 | -0.987 | -0.4559 | Yes |
| 55 | HIST1H1E |  |  | 2202 | -0.992 | -0.4408 | Yes |
| 56 | SGO2 |  |  | 2203 | -1.003 | -0.4256 | Yes |
| 57 | SMC2 |  |  | 2205 | -1.006 | -0.4108 | Yes |
| 58 | EZH2 |  |  | 2207 | -1.017 | -0.3958 | Yes |
| 59 | DHFR |  |  | 2212 | -1.040 | -0.3818 | Yes |
| 60 | NUDT1 |  |  | 2214 | -1.054 | -0.3662 | Yes |
| 61 | CLSPN |  |  | 2216 | -1.060 | -0.3506 | Yes |
| 62 | H2AFZ |  |  | 2220 | -1.090 | -0.3354 | Yes |
| 63 | HMGB1 |  |  | 2221 | -1.116 | -0.3185 | Yes |
| 64 | CDK1 |  |  | 2225 | -1.153 | -0.3023 | Yes |
| 65 | PTTG1 |  |  | 2226 | -1.198 | -0.2841 | Yes |
| 66 | ANP32E |  |  | 2227 | -1.200 | -0.2659 | Yes |
| 67 | UBE2S |  |  | 2229 | -1.205 | -0.2481 | Yes |
| 68 | HIST2H2AC |  |  | 2231 | -1.231 | -0.2299 | Yes |
| 69 | DBF4 |  |  | 2232 | -1.247 | -0.2109 | Yes |
| 70 | HMGN2 |  |  | 2233 | -1.267 | -0.1917 | Yes |
| 71 | SMC4 |  |  | 2236 | -1.281 | -0.1731 | Yes |
| 72 | ATAD2 |  |  | 2238 | -1.381 | -0.1526 | Yes |
| 73 | PRC1 |  |  | 2240 | -1.397 | -0.1319 | Yes |
| 74 | HMGB2 |  |  | 2241 | -1.469 | -0.1095 | Yes |
| 75 | BIRC5 |  |  | 2242 | -1.469 | -0.0872 | Yes |
| 76 | HIST1H1C |  |  | 2243 | -1.485 | -0.0647 | Yes |
| 77 | PCLAF |  |  | 2245 | -1.516 | -0.0421 | Yes |
| 78 | HIST1H4C |  |  | 2253 | -2.984 | -0.0000 | Yes |
Table: GSEA details [plain text format]

  

Fig 2: NB\_KI\_CLUSTER5: Random ES distribution      
 Gene set null distribution of ES for **NB\_KI\_CLUSTER5**
